# Comprehensive Metabolome and Volatilome Analyses in Eggplant and Tomato Reveal Their Differential Responses to *Tuta absoluta* Infestation

**DOI:** 10.3389/fpls.2021.757230

**Published:** 2021-11-03

**Authors:** Limin Chen, Xiaowei Li, Jinming Zhang, Tianjun He, Jun Huang, Zhijun Zhang, Yeyang Wang, Muhammad Hafeez, Shuxing Zhou, Xiaoyun Ren, Youming Hou, Yaobin Lu

**Affiliations:** ^1^State Key Laboratory for Managing Biotic and Chemical Threats to the Quality and Safety of Agro-Products, Institute of Plant Protection and Microbiology, Zhejiang Academy of Agricultural Sciences, Hangzhou, China; ^2^State Key Laboratory of Ecological Pest Control for Fujian and Taiwan Crops, Key Lab of Biopesticide and Chemical Biology, Ministry of Education & Fujian Key Laboratory of Insect Ecology, College of Plant Protection, Fujian Agriculture and Forestry University, Fuzhou, China; ^3^Integrated Plant Protection Center, Lishui Academy of Agricultural and Forestry Sciences, Lishui, China

**Keywords:** electroantennography, biocontrol approach, host preference, plant defense metabolites, volatiles

## Abstract

The South American tomato pinworm, *Tuta absoluta*, is one of the most destructive insect pests in Solanaceae crops, particularly in tomatoes. Current methods of management have proven somewhat effective but still require a more efficacious management strategy to limit its havoc on crop yield. Tomato is much more predisposed to *T. absoluta* as compared with other plants such as eggplants, but the underlying causes have not been fully determined. We conducted this study to unravel the volatile organic compounds (VOCs) and primary/secondary metabolites that account for the differential response of tomatoes and eggplants to *T. absoluta* infestation. We performed widely targeted comparative metabolome and volatilome profiling by ultraperformance liquid chromatography–tandem mass spectrometry (UPLC-MS/MS) and headspace solid-phase microextraction coupled to gas chromatography–mass spectrometry (HS-SPME/GC-MS), respectively, on eggplants and tomatoes under control and *T. absoluta* infestation conditions. Overall, 141 VOCs and 797 primary/secondary metabolites were identified, largely dominated by aldehyde, alcohols, alkanes, amine, aromatics, a heterocyclic compound, ketone, olefin, phenol, and terpenes. Most of the VOCs and primary/secondary metabolites from the terpene class were largely differentially regulated in eggplants compared with tomatoes. Eggplants emitted several compounds that were lower or completely absent in tomatoes either under control conditions or after *T. absoluta* infestation. The results from an electroantennogram showed that 35 differentially accumulated VOCs could elicit female *T. absoluta* response, implying that these volatile compounds significantly alter the behavior of this pest. These findings demonstrated that differentially accumulated metabolites and volatile compounds play major roles in eggplant resistance to *T. absoluta* infestation as these compounds were regulated upon attack by *T. absoluta*. Our findings can assist in integrated pest management efforts by developing appropriate control measures against *T. absoluta* in Solanaceae production.

## Introduction

The South American tomato pinworm [*Tuta absoluta* (Meyrick)] has become one of the most destructive pests in tomato production worldwide (Bawin et al., 2016; Zhang et al., 2020). *Tuta absoluta* belongs to the *Gelechiidae* family in the order Lepidoptera (Zhang et al., 2020) and was first described in South America in 1917, which caused extensive damage to tomatoes and other plants (Biondi et al., 2018; Cherif and Verheggen, 2019; Tarusikirwa et al., 2020). The pest has since spread widely to ~110 countries in Africa, America, Asia, Europe, and the Middle East through the large-scale importation of tomatoes in those countries (Desneux et al., 2010; Biondi et al., 2018; Mansour et al., 2018; Guimapi et al., 2020). *Tuta absoluta* feeds, overwinters, and breeds on a broad range of hosts such as eggplant, potato, nightshade, tomato, and tobacco (Silva et al., 2021). Tomato is reported to be the most preferred host (Shah et al., 2015; Negi et al., 2018). The larvae damage the leaves, buds, stems, and fruits, with a detrimental effect on fruit yield and quality (Torres et al., 2001; Desneux et al., 2010). The economic effect of this pest in tomato production and yield cannot be overemphasized (Campos et al., 2017). The poor management of *T. absoluta* infestations can lead to 80–100% crop loss (Maluf et al., 2010).

Solanaceae vegetables, such as tomatoes (*Solanum lycopersicum* L.) and eggplants (*Solanum melongena* L.) are multi-nutritive vegetables that are cultivated all over the world and consumed in both fresh and processed forms (Kavanaugh et al., 2007; Gerszberg et al., 2015). However, biotic factors such as insects and pathogens are significant impediments to their long-term development (Shah et al., 2015). Tomato and eggplant cultivation under field and greenhouse conditions are rapidly expanding into new regions in China (Desneux et al., 2010; Biondi et al., 2018; Cherif and Verheggen, 2019; Zhang et al., 2020). Although researchers and the government have attempted to limit the spread of *T. absoluta* in China by working together, the pest was found in the Xinjiang region of China in 2017 (Zhang et al., 2020). In 2018, the damage of this pest has also been reported in protected-field fresh marketing tomatoes in Lincang, Yunnan province (Cherif and Verheggen, 2019; Zhang et al., 2020). Recent studies have found that *T. absoluta* is spreading at 800 km each year (Cherif and Verheggen, 2019; Li Xiaowei, 2019). In the light of these new findings, specifically in terms of migratory patterns, a more focused tracking of tomato-growing areas is necessary throughout China; otherwise, tomatoes elsewhere in the country will lose their resistance to the pest.

There have been several efforts to monitor the global invasions of *T. absoluta* over the past decade (Campos et al., 2017; Han et al., 2018; Li et al., 2021). Chemical control is the primary measure to manage *T. absoluta* in both native and invaded areas (Silva et al., 2018; Langa et al., 2021). However, the large use of chemical pesticides led to environmental pollution and food safety challenges (Machekano et al., 2018). Consequently, various pesticide-resistant strains have emerged in different locations (Zappal et al., 2013; El-Arnaouty et al., 2014; Biondi et al., 2015). Alternative methods of management, such as using its natural enemies (El-Arnaouty et al., 2014; Biondi et al., 2015; Silva et al., 2018; Langa et al., 2021) and pheromones, have proven somewhat effective in *T. absoluta* population suppression (Aksoy and Kovanci, 2016; Mirhosseini et al., 2019), but still requires a more efficacious management strategy to limit its havoc on crop yield.

Recent advances have been made in tomatoes through the use of genetic and genomic techniques to limit insect damages (Comparative biochemical and transcriptome analyses in tomato and eggplant reveal their differential responses to Tuta absoluta infestation | Mendeley; Cocco et al., 2013; Zhou et al., 2020; Chen et al., 2021). However, current research on the South American tomato pinworm mainly focuses on invasion status, host range, pesticide resistance, and natural enemy control. There are relatively limited research reports on the metabolites induced or repressed by *T. absoluta* infestation and on host preference and interaction mechanism at the molecular level (Rostami et al., 2020). The survival and feeding preference of herbivorous insects are partly controlled by plant metabolites. An attack or wounding by *T. absoluta* larvae often induces specific metabolic variations in plants to regulate feeding, mating, and oviposition (Cha et al., 2008; Gripenberg et al., 2010; Fatouros et al., 2016). A study on tomatoes revealed that the interaction between brassinosteroids and jasmonates is needed to counteract attacks by *T. absoluta* (Campos et al., 2009). Phytohormones such as auxin, brassinosteroids, ethylene, gibberellin, jasmonates, and salicylic acid are reported to act synergistically or antagonistically to activate signal transduction upon insect feeding or wounding (Howe and Jander, 2008; War et al., 2011). Besides the effects of plant metabolites on survival and feeding preference, the preference of herbivorous insects for host plants mainly depends on the choice of adult oviposition and the suitability of larvae on host plants (War et al., 2011). Plant volatile organic compounds (VOCs) do not only play an important role in the interaction between plants and insects but are important chemo-information for phytophagous insects to locate host plants (Proffit et al., 2011; De Backer et al., 2015; Anastasaki et al., 2018). The role of plant volatiles in integrated pest management has increasingly gained research attention (Anastasaki et al., 2018). After a tomato is infested by South American tomato pinworm larvae, the plant can trigger herbivore-induced plant volatiles (HIPVs) by releasing (z)-3-hexen-1-ol, methyl salicylate, and indole-3 acetic acid to halt further egg-laying (Leitner et al., 2005). Similarly, in related studies on oviposition-inducing volatiles (OIPVs), it was found that the oviposition behavior of *T. absoluta* adults can induce the production of the tomato volatiles (z)-3-hexen-1-ol. In a study on the interaction between two natural enemies of *T. absoluta*, it was found that tomato plants with natural enemies can release the volatile octyl acetate to attract the egg-laying larvae of *T. absoluta* adults (Howe and Jander, 2008). Again, studies have shown that the compositions of different tomato volatiles are also different, and there are certain differences in the attracting ability of tomato leaf miners (War et al., 2011; Rostami et al., 2020).

Researchers have been investigating the various mechanisms of *T. absoluta* infestations in tomatoes and other species (Desneux et al., 2010; Cherif and Verheggen, 2019; Silva et al., 2021). In our earlier studies, the *T. absoluta* growth and survival rate in tomatoes was found to be substantially higher than in eggplants (Li Xiaowei, 2019). The mechanism that causes *T. absoluta* to prefer tomatoes over eggplants has been partly attributed to the differences in the behavioral selection and fitness of different host plants by herbivorous insects (Sankarganesh et al., 2017). Thus, the females of herbivorous insects lay more eggs on host plants that are conducive to the growth and development of their offspring, making the offspring more attracted to the host plants (Arnó et al., 2019). These differences in the behavioral selection and fitness of herbivorous insects to host plants mainly depend on the differential production of metabolites and volatiles by different host plants (Sylla et al., 2019). Some herbivorous insects can secrete corresponding detoxification enzymes for the degradation of toxic and harmful substances produced by host plants (Cherif and Verheggen, 2019). The current study profiled the metabolites and volatiles induced in tomatoes and eggplants in response to *T. absoluta* infestation. We also profiled the key plant metabolites that cause differences in the behavioral selection and fitness of *T. absoluta* and assessed the interaction between *T. absoluta* in tomatoes and eggplants and their effect on the synthesis of key plant metabolites. Theoretically, the study provided insights into host preferences by *T. absoluta*, which enriches our understanding of *T. absoluta* invasion biology and, in practice, as a guide for the development and application of plant-derived attractants and repellents against *T. absoluta*.

## Results

### Targeted Volatilome and Metabolome Profiling of Healthy and *T. absoluta* Infested Leaf Samples in Tomatoes and Eggplants

**Figure 1:**
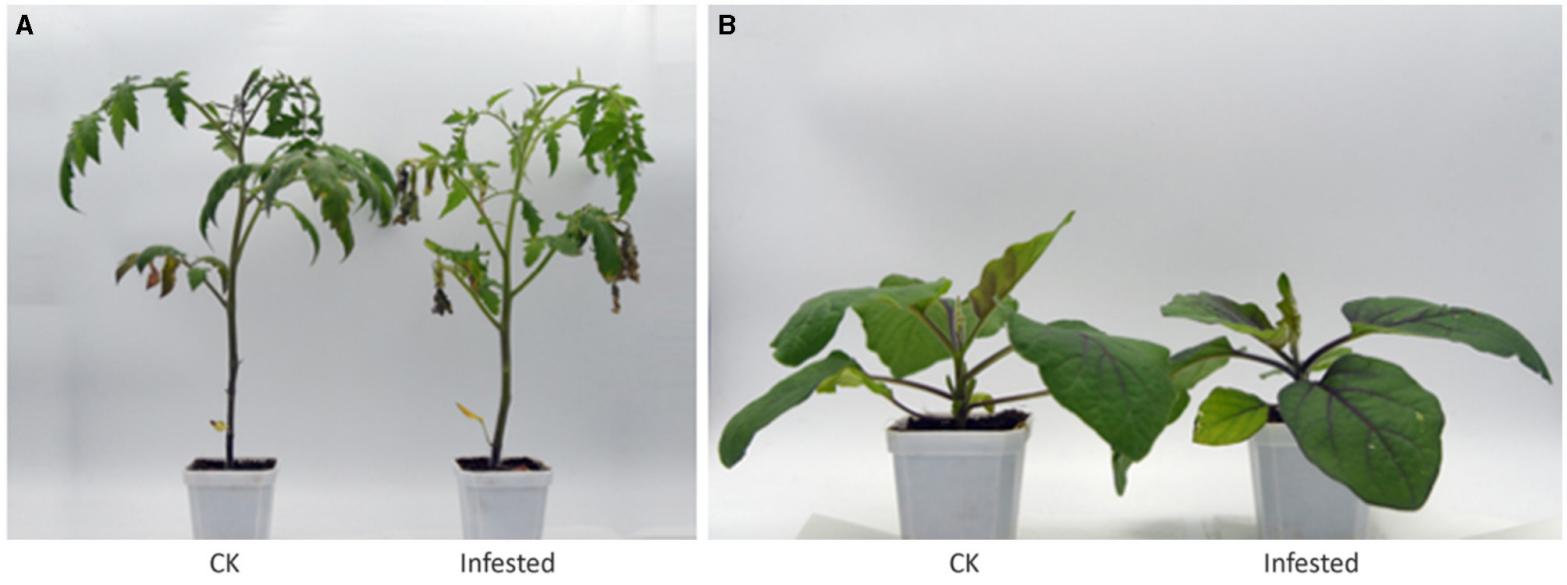
Healthy and infested plants of tomato **(A)** and eggplant **(B)**. The infested plants were obtained in a growth chamber 48 h post-*T. absoluta* infestation. CK, control plant.

This study was undertaken to identify the metabolites and volatiles that may account for the *T. absoluta* preference for tomatoes than eggplants as observed in a previous study (Li et al., 2019). The two species of Solanaceae were inoculated with *T. absoluta* in a growth chamber (Figure 1). Tomatoes exhibited severe symptoms of leaf yellowing and other symptoms associated with *T. absoluta* infestation (Mutamiswa et al., 2017). The majority of the fallen *T. absoluta* mature larvae were observed under the tomato plants, an indication that *T. absoluta* thrived and multiplied more easily in tomatoes than in eggplants. We sampled the two species under control conditions (healthy leaf free from *T. absoluta* infestation) and *T. absoluta-*infested conditions to quantitatively profile VOCs and primary/secondary metabolites with headspace solid-phase microextraction (HS-SPME) coupled with gas chromatography–mass spectrometry (GC-MS) and ultra-performance liquid chromatography–tandem mass spectrometry (UPLC-MS/MS).

**Figure 2:**
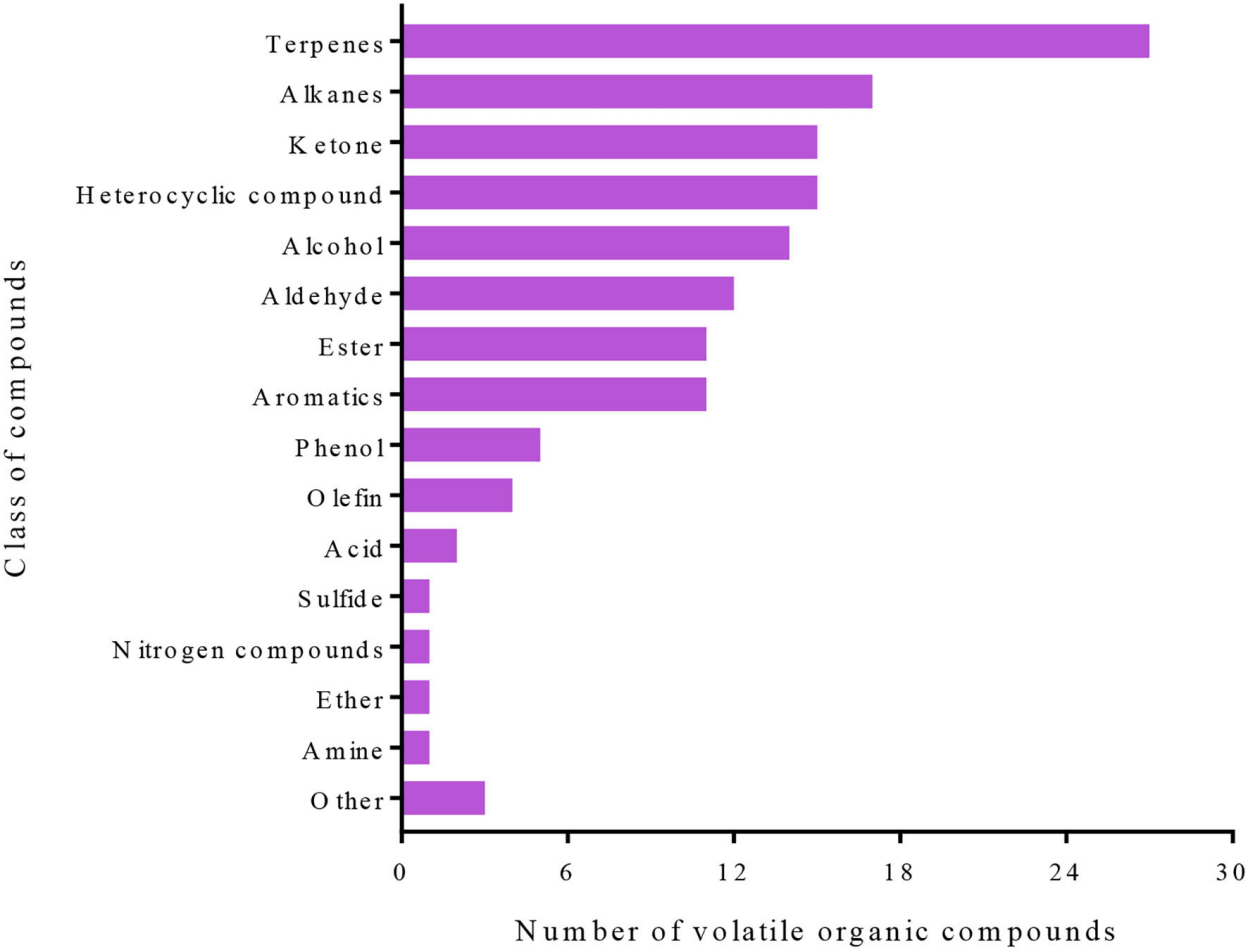
Classes of volatile organic compounds identified in the control and infested leaves of tomatoes and eggplants.

**Figure 3:**
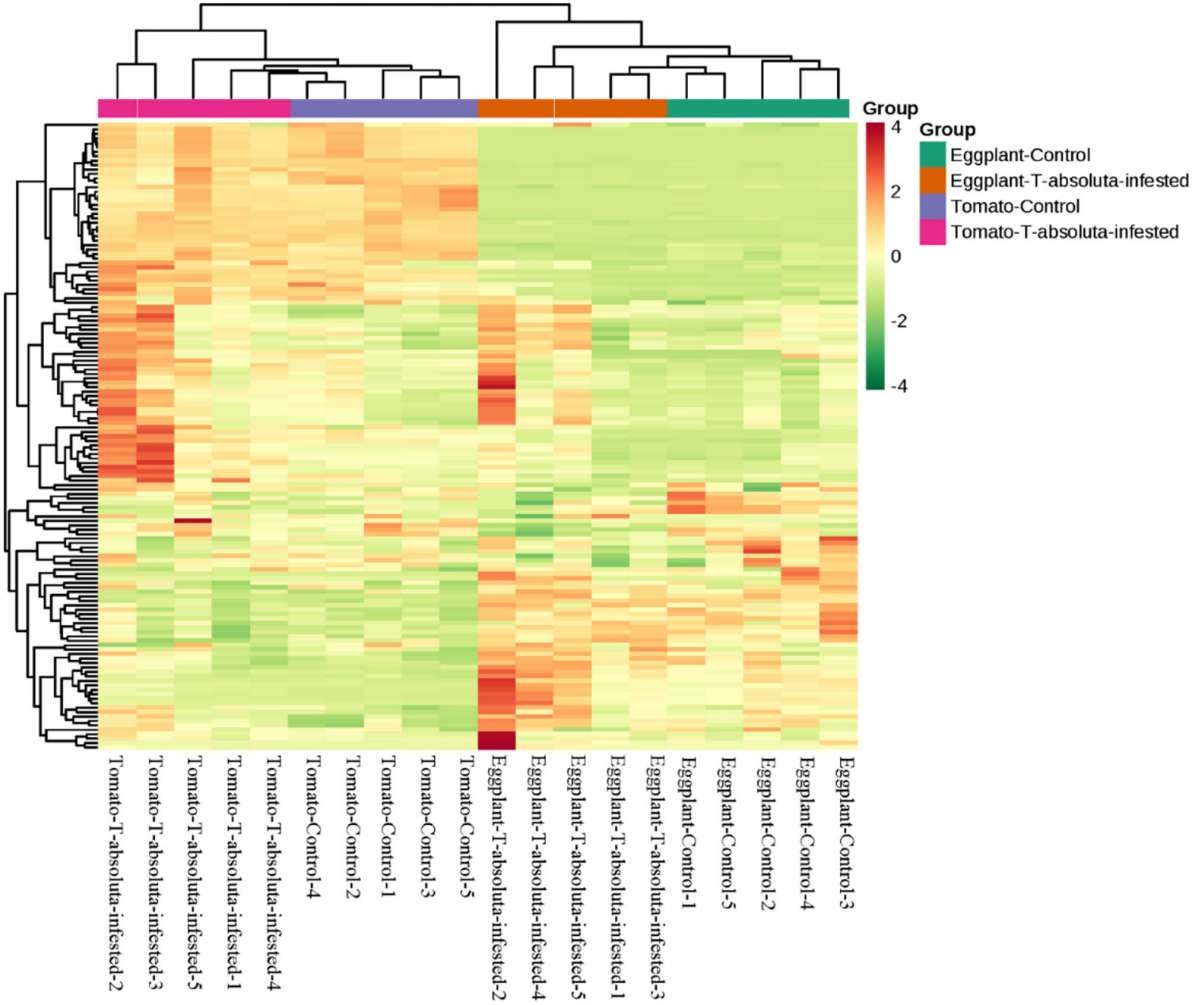
Hierarchical clustering based on the ion intensity of volatile organic compounds from leaves of tomato and eggplant. All analyses were done in quintuplicate: Eggplant-Control (1–5), Eggplant-*T. absoluta* infested (1–5), Tomato-Control (1–5), and Tomato-*T. absoluta* infested (1–5).

This study has identified 141 VOCs, which were predominantly from terpenes, alkanes, ketone, heterocyclic compounds, alcohol, aldehyde, aromatics, phenol, and olefin classes that were detected in the four groups that had been evaluated (Figure 2). A hierarchical clustering analysis based on the ion intensities of the VOCs detected in the four groups (Tomato-Control, Tomato-*T. absoluta* infested, Eggplant-Control, and Eggplant-*T. absoluta* infested) clustered them into two main VOC clusters (Figure 3). Each cluster consisted of only one species of Solanaceae with its biological repeats (either control or *T. absoluta* infested) sub-clustered together. The results indicated that there are significant differences in the volatile profiles between tomatoes and eggplants either in control conditions or upon infestation by *T. absoluta*.

**Figure 4:**
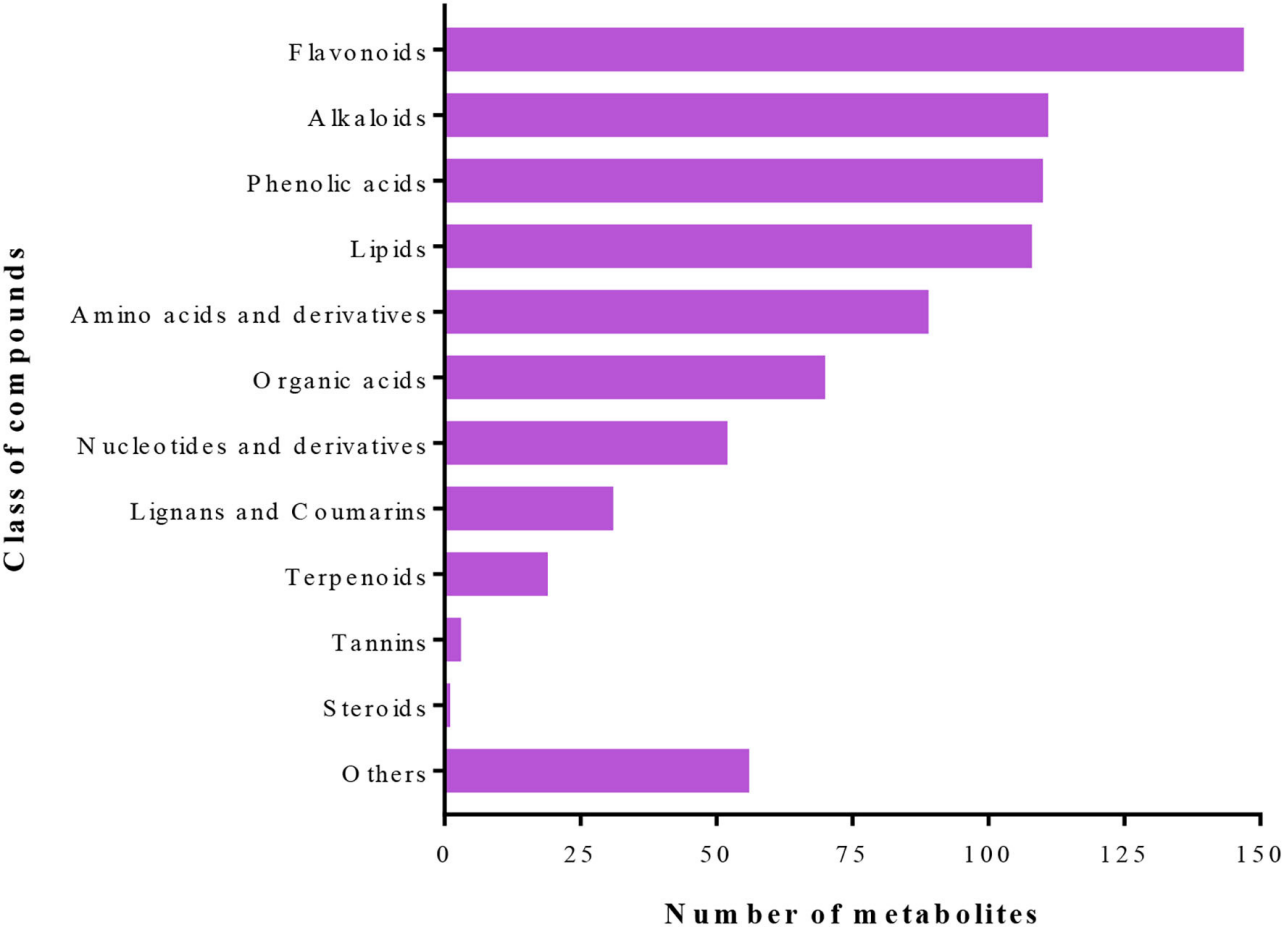
Class of primary/secondary metabolites detected in the leaves of control and infested tomatoes and eggplants.

**Figure 5:**
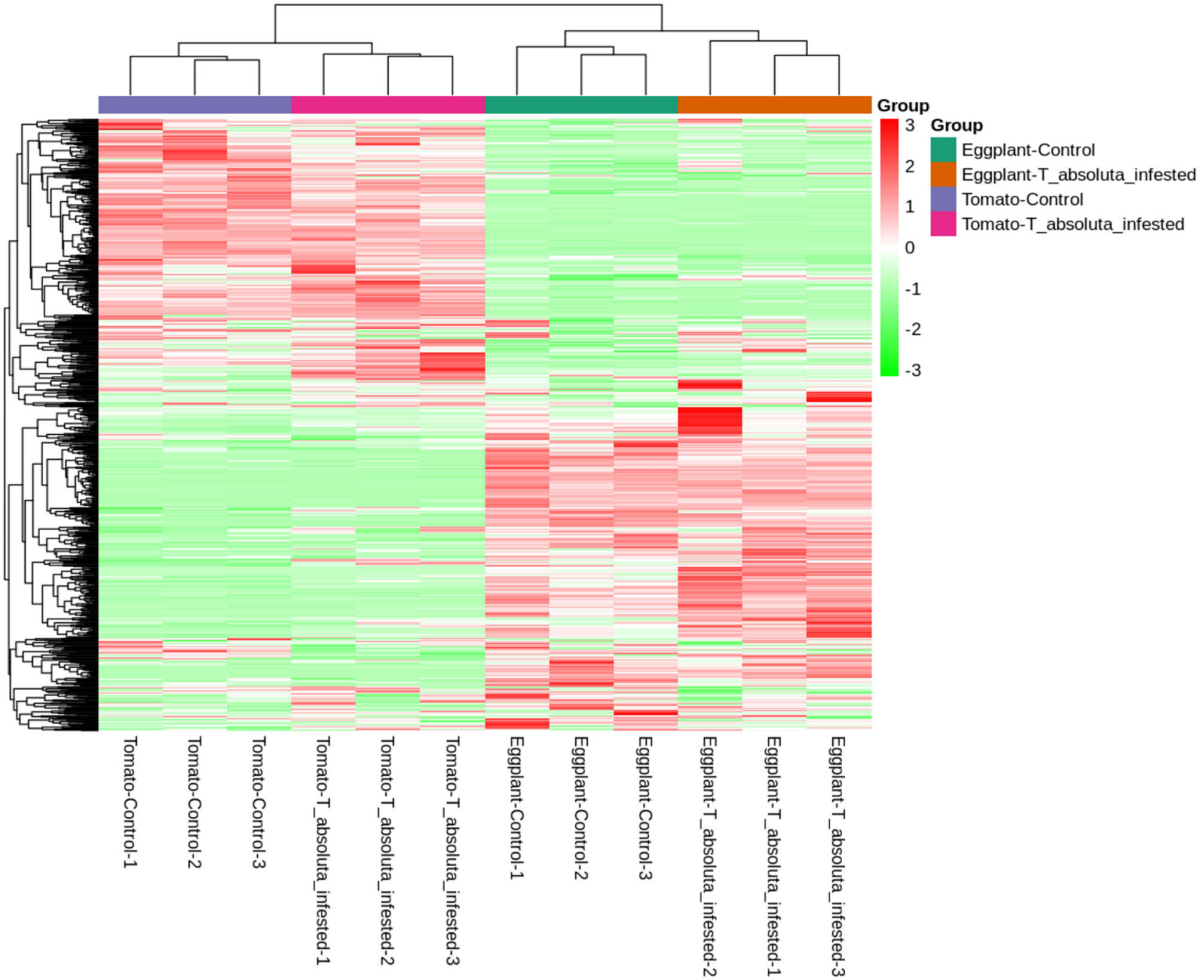
Hierarchical clustering based on the ion intensity of primary/secondary metabolites from the leaves of tomatoes and eggplants. All analyses were done in triplicates: Eggplant-Control (1–3), Eggplant-*T. absoluta* infested (1–3), Tomato-Control (1–3), and Tomato-*T. absoluta* infested (1–3).

A total of 797 primary/secondary metabolites were identified among the four groups. These metabolites were from flavonoids, alkaloids, phenolic acids, lipids, amino acids and derivatives, organic acids, nucleotides and derivatives, lignans and coumarins, terpenoids, tannins, steroids, and others (Figure 4). Interestingly, one steroid compound (Aculeatiside A) was detected only in eggplants under the two conditions. We subjected the metabolite ion intensities of the four groups to hierarchical clustering analysis, resulting in two main clusters (Figure 5). Cluster I comprised solely of tomatoes and cluster II consisted of samples from eggplants (Figure 5). The three biological replicates of tomatoes or eggplants formed one sub-cluster in each cluster. This trend indicated that a limited number of primary/secondary compounds were induced by *T. absoluta* infestation. The clustering patterns of the metabolomes and volatilomes of biological repeats in the same sub-cluster pinpointed the nature of compounds and the repeatability of compounds in tomato and eggplant under either control or *T. absoluta* infestation conditions (Figures 3, 5).

### Differentially Accumulated Volatile Compounds Identified in the Pairwise Groups

**Figure 6:**
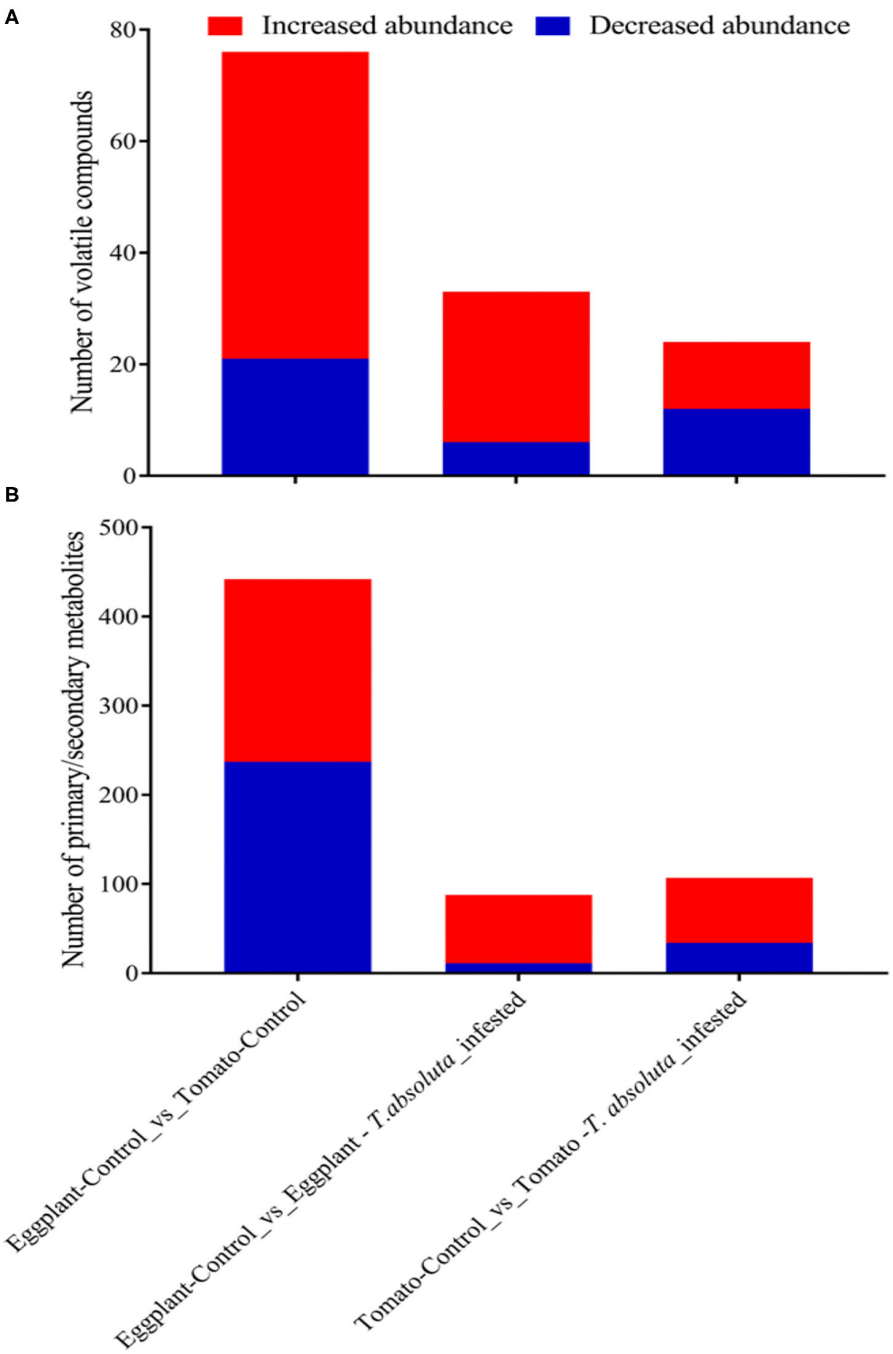
Differentially accumulated compounds in the leaves of tomatoes and eggplants under two different conditions. Discriminant analysis of orthogonal partial least squares with a threshold of log2FC ≥ 1 and variable importance in projection ≥ 1. **(A)** Differentially accumulated volatile organic compounds. **(B)** Differentially accumulated primary/secondary metabolites.

We further applied a discriminant analysis of orthogonal partial least squares (OPLS-DA) at a threshold of log2FC ≥ 1 and variable importance in projection (VIP) ≥ 1 to identify differentially accumulated volatile organic compounds (DAVOCs) and differentially accumulated metabolites (DAMs) among the four groups in pairwise comparisons. Overall, 96 unique DAVOCs were detected among Eggplant-Control vs. Tomato-Control, Eggplant-Control vs. Eggplant-*T. absoluta* infested, and Tomato-Control vs. Tomato-*T. absoluta* infested from 16 classes of compounds (Supplementary Tables 1–3). The extent of the occurrence and accumulation of these DAVOCs varied among the four treatments (Supplementary Figures 1A–D). There were 76 VOCs detected between Eggplant-Control and Tomato-Control; of these, 21 VOCs accumulated lower in Eggplant-Control and 55 VOCs accumulated higher in Tomato-Control (Supplementary Table 1A). This indicated that most of the VOCs found in the tomato headspace were more abundant than in eggplants (Figure 6A). For instance, 1 alkane (1,4,7,-Cycloundecatriene, 1,5,9,9-tetramethyl-, Z,Z,Z), 1 ester [2-Butenoic acid, 3-hexenyl ester, (E,Z)], 2 heterocyclic compounds [2,2'-Isopropylidenebis(5-methylfuran) and Pyrazine, Furfuryl Methyl Disulfide], and 10 terpenes [Nerolidol 2; gamma.-Murolene; 6-Isopropyl-1,4-dimethylnaphthalen; 10,10-Dimethyl-2,6-dimethylenebicyclo[7.2.0]undecane; 3a,7-Methano-3aH-cyclopentacyclooctene, 1,4,5,6,7,8,9,9a-octahydro-1,1,7-trimethyl-, [3aR-(3a.alpha.,7.alpha.,9a.beta.)]; alpha.-Cuprenene;α-thujene; (3E,7E)-4,8,12-Trimethyltrideca-1,3,7,11-tetraene;(+)-4-Careneand Cyclohexene, 4-ethenyl-4-methyl-3-(1-methylethenyl)-1-(1-methylethyl)-, (3R-trans)] were more abundant in uninfected tomatoes (Supplementary Table 1). The increased volatiles in tomatoes might contribute to the *T. absoluta* preference for tomatoes over eggplants. On the other hand, some volatiles, such as trans,cis-2,6-non-adienal, trans,trans-2,4-non-adienal, hexadecane, γ-caprolactone, and 2,2,4,4,6,8,8-heptamethylnonane, were more abundant in uninfected eggplants, and these volatiles might have repellent activities to *T. absoluta*. To identify volatile compounds which may act as stimulants for host location and damage by *T. absoluta* on tomatoes, we compared the volatiles found in Tomato-Control with those found in *T. absoluta*-infested tomato. A total of 24 DAVOCs, comprising 12 each for decreased or increased in abundance (Figure 6A), were identified. From these, four terpenes (Nerolidol 2; beta-Cyclocitral; 1,3-Cyclohexadiene-1-carboxaldehyde, 2,6,6-trimethyl, and beta.-iso-Methyl ionone), one ketone (Cyclohexanone, 2,2,6-trimethyl), one heterocyclic compound (Furan, 2-pentyl), two ester [2-Butenoic acid, 3-hexenyl ester, (E,Z) and (E)-Hex-3-enyl (E)-2-methylbut-2-enoate], two aldehydes (Benzaldehyde and 2-Ethylbenzaldehyde), and two alcohols (Cyclohexanol, 2,6-dimethyl, and 1-Octen-3-ol) could be potential attractants and/or stimulants of *T. absoluta* in tomatoes (Supplementary Table 3).

We further compared Eggplant-Control and *T. absoluta* infested eggplants. There were 27 volatiles that increased in abundance upon infestation (Figure 6A). Of these, one Terpene (Nerolidol 2), one Olefin (1,3-Cyclopentadiene, 5,5-dimethyl-1,2-Dipropyl), and one ester [2-Butenoic acid, 3-hexenyl ester, (E,Z)] were detected upon infestation (Supplementary Table 2). In addition, 25 volatiles accumulated 1.21–47.55 times higher in Eggplant-*T. absoluta* infested than Eggplant-Control (Supplementary Table 2). On the contrary, three alcohols (1-Heptanol, 1-Nonanol, and 1-Nonanol), one ester (Formic acid, octyl ester), one Olefin (1-Undecene, 9-methyl), and one Ketone (3-Nonen-5-one) accumulated 1.36–1.69 times higher in Eggplant-Control than Eggplant-*T. absoluta* infested (Supplementary Table 2). These volatiles may be antifeedants for *T. absoluta* in eggplants.

### Differentially Accumulated Primary/Secondary Metabolites Identified in the Pairwise Groups

There were 442 primary/secondary metabolites found to be accumulated differentially between Eggplant-Control and Tomato-Control (Figure 6B; Supplementary Table 4). These were mostly composed of flavonoids, alkaloids, phenolic acids, and lipids (Supplementary Table 4). Additionally, these compounds mostly accumulated higher in Eggplant-Control than in Tomato-Control. For example, 55 alkaloids, 72 flavonoids, 15 lignans and coumarins, 55 lipids, 36 phenolic acids, 12 organic acids, 11 nucleotides and derivatives, 10 amino acids and derivatives, 2 terpenoids, and 6 other compounds were highly accumulated in Eggplant-Control or were completely absent in Tomato-Control (Supplementary Table 4). It is worth mentioning that the presence of one steroid compound, namely, “Aculeatiside A,” in Eggplant-Control, which was completely absent in Tomato-Control, was found. This steroid compound may be a repellent or an antifeedant and antidigestive to *T. absoluta* in eggplants compared with tomatoes. Conversely, 205 compounds accumulated higher in Tomato-Control or were completely absent in Eggplant-Control. Among these, alkaloids (dehydrocommersonine, β1-Tomatine, Lycoperoside A, and Esculeogenin A-27-O-rhamnoside), amino acids and derivatives (S-(Methyl)glutathione), Cyclo (Phe-Glu), O-Phospho-L-serine and N-Acetyl-L-Tryptophan, flavonoids (Tricin-5-O-Glucoside, Sieboldin, Quercetin-3-O-(2”-O-arabinosyl), rutinoside and Cyanidin-3-O-(6”-O-p-Coumaroyl)glucoside), lignans and Coumarins [Fraxetin (7,8-Dihydroxy-6-methoxycoumarin), 7-Methoxycoumarin, Daphnetin, and Olivil-4'-O-glucoside], organic acids (2-Hydroxyphenylacetic acid, abscisic acid, muconic acid, and (-)-Jasmonoyl-L-Isoleucine), phenolic acids (rhododendron; 3,4-Dihydroxybenzeneacetic acid; homogentisic acid; oleoside 11-methyl ester) were highly accumulated in Tomato-Control or were completely absent in Eggplant-Control (Supplementary Table 4). These compounds may be the basis for the higher preference of *T. absoluta* for tomatoes. These results suggested that eggplants induced several primary/secondary metabolites to enhance their defense against *T. absoluta* or repel *T. absoluta* from damaging the leaves. The absence or presence of some metabolites in the Tomato-Control compared with the Eggplant-Control are likely to make tomatoes more prone to *T. absoluta* infestation than eggplant.

Eggplant-Control in relation to *T. absoluta*-infested eggplants had 88 primary/secondary metabolites with differential accumulation comprising 11 decreased and 77 increased metabolites in abundance upon infestation, respectively (Figure 6B; Supplementary Table 5). This implied that the increased abundance of several metabolites in eggplants was mainly to ward off or prevent the survival and multiplication of *T. absoluta*. Eight of such compounds exclusively detected upon infestation included two alkaloids [N-(4-O-(Glucosyl)-E-feruloyl)-tyramine and esculeogenin A-27-O-rhamnoside], two amino acids and derivatives [O-Phospho-L-serine and S-(Methyl)glutathione], two flavonoids [6-Hydroxykaempferol-3-O-rutin-6-O-glucoside and Quercetin-3-O-apiosyl (1 → 2) galactoside], one lignans and coumarins (7-Methoxycoumarin), and one organic acid (Methyl jasmonate). In contrast to these, one flavonoid (Genistein-7-O-galactoside) and one nucleotide and derivative (2-Aminopurine) were detected exclusively in Tomato-Control (Supplementary Table 5).

By comparing the metabolome of Tomato-Control with *T. absoluta*-infested tomato, a total of 107 primary/secondary metabolites that were differentially accumulated (34 downregulated and 73 upregulated) were identified (Figure 6B; Supplementary Table 6). This implied that *T. absoluta*-infested tomatoes induce primary/secondary metabolites that contribute to the survival and multiplication of *T. absoluta*. Eight of such compounds exclusively detected upon infestation include two alkaloids [3,5-Bis(3-methoxy-4-hydroxyphenyl)-2,3-dihydro-2(1H)-pyridinone and Lycibarbarspermidine I], two flavonoids (Peonidin-3-O-glucoside and 6”-O-Malonylgenistin), two lignans and coumarins (Lariciresinol-4'-O-glucoside and Syringaresinol), one lipid (LysoPE 20:5), and one organic acid (2-Aminoethanesulfonic acid). In contrast to these, five flavonoids [Apigenin-6-C-rhamnoside, Patuletin-7-O-rutinoside, Eriodictyol-7-O-glucoside, Delphinidin-3-O-(2”'-O-p-coumaroyl) rutinoside-7-O-glucoside and Cyanidin-3-O-glucoside (Kuromanin)], two terpenoids [Betulinic acid and Medicagenic acid-3-O-glucosyl-(1,6)-glucosyl-(1,3)-glucoside], one phenolic acid (Isochlorogenic acid C), one organic acid (2-Aminoethanesulfinic acid), and one amino acid and derivative (γ-Glu-Cys) were detected exclusively in Tomato-Control (Supplementary Table 6). These compounds may have contributed to the preference of *T. absoluta* for tomatoes.

### Electroantennographic Response of *T. absoluta* Females to VOCs

**Figure 7:**
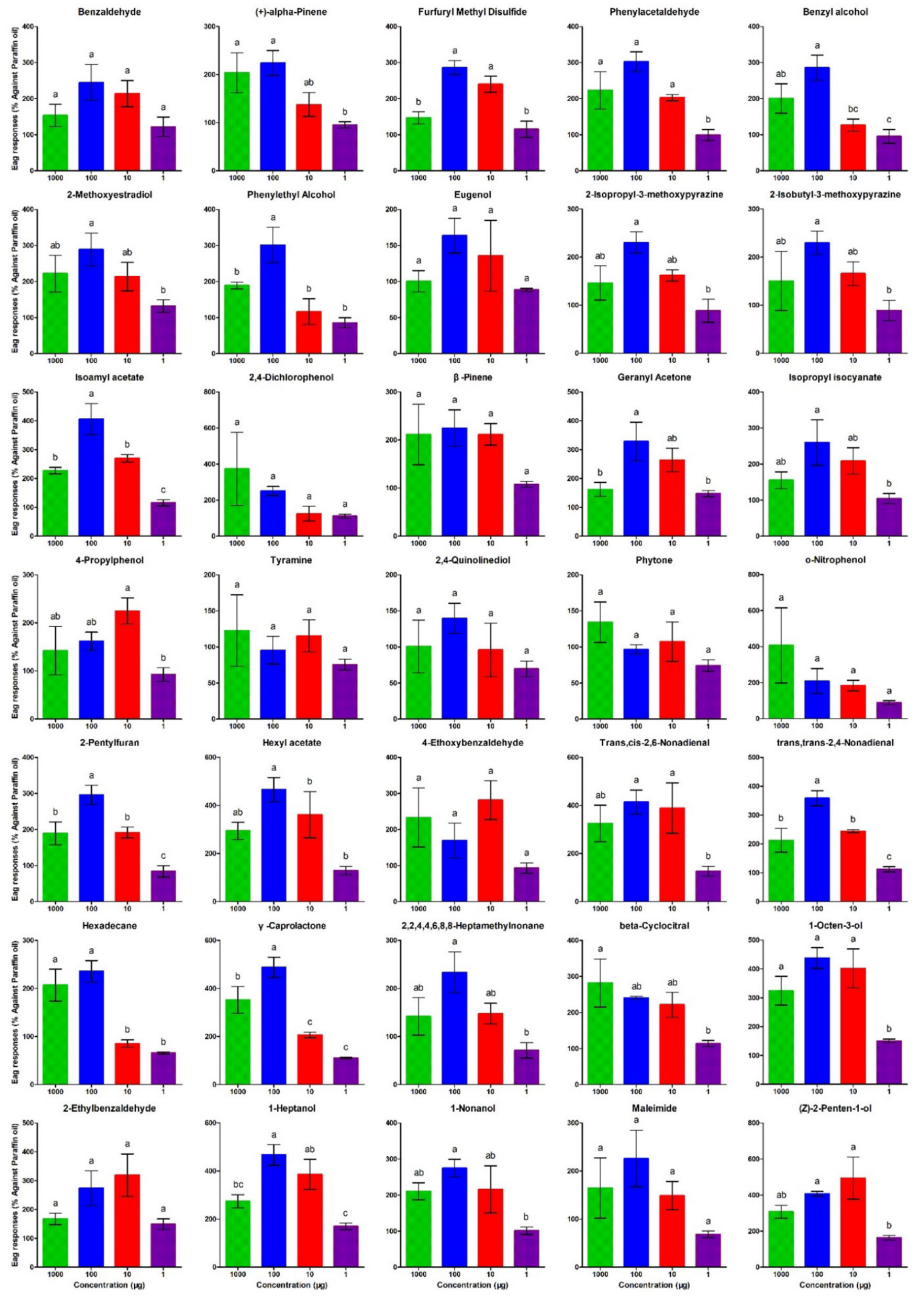
Electroantennographic (EAG) response of *T. absoluta* females to volatile compounds. The compound names are displayed on top of each chart. The bar represents the mean of the triplicate reading from the EAG experiment. Error bars represent the SEM (± SEM). Bars for each compound with a common alphabet displayed on top indicate the significant difference with Turkey's Highest Significant Difference at *P* < 0.05.

To verify whether the VOCs identified in this study could influence *T. absoluta* behavior, the electroantennogram (EAG) response of *T. absoluta* to 35 VOCs was investigated. The results showed that all 35 compounds could elicit electrophysiological responses in the antennae of female *T. absoluta* (Figure 7). Of note, 2-Methoxyestradiol, Isoamyl acetate, Geranyl Acetone, Hexyl acetate, Trans, cis-2,6-Non-adienal, γ-Caprolactone, 1-Octen-3-ol, 1-Heptanol, and (Z)-2-Penten-1-ol elicited the highest EAG responses among all ligands in females.

## Discussion

It is well documented that *T. absoluta* attacks over 26 different host plant species with much preference for solanaceous species, with tomato as one of the most preferred hosts (Figure 1) (Özgökçe et al., 2016; Wellington et al., 2019; Dong and Lin, 2021). However, knowledge on the basis for this preference is limited since most of the previous studies profiled only VOCs (Anastasaki et al., 2018; Parasitoids et al., 2019) or primary/secondary metabolites (de Falco et al., 2019). Combining VOCs and primary/secondary metabolites in this present study offered the potential for identifying compounds that may have been lost/altered during domestication and breeding efforts (Alseekh et al., 2021), which may be responsible for the differential response between the two solanaceous species for the possible development of eco-friendly management strategies against *T. absoluta* in tomatoes.

Plants produce several compounds, both VOCs and primary/secondary metabolites, in response to insect/pest attacks (Bruce et al., 2005). These compounds act as repellents, deterrents, attractants, antinutrients, and anti-digestives either before/after attack or both. Terpenoids/terpenes and other VOCs released from plants in response to insect attacks allow parasitoids and predators to differentiate between infested and non-infested plants by assisting in the location of a host or prey (Paré P. W., 2008). In this study, 141 VOCs and 797 primary/secondary metabolites have been identified, which were largely flavonoids, alkaloids, phenolic acids, lipids, amino acids and derivatives, organic acids, nucleotides and derivatives, lignans and coumarins, terpenoids, tannins, steroids, and several other minor classes of compounds (Figures 2, 4).

We observed that eggplants emitted several compounds that were lower or completely absent in tomatoes either under control or *T. absoluta*-infestation conditions and vice versa (Supplementary Tables 1–6). It has been reported that the cytosolic mevalonate pathway is key in defense against insects, which starts with farnesyl diphosphate (FPP) but remains the same in insects and plants. Insects, through FPP, form a juvenile hormone that plays a role in the regulation of molting (Dubey, 2013). Steroids and sterols are products of terpenoid precursors, which are lacking in insects but can only be utilized by extracting the needed precursors for cholesterol and other steroids from their food (Belles and Martin, 2005). Therefore, the only steroid compound, namely, Aculeatiside A, potentially makes eggplant unattractive to *T. absoluta* (Figure 4; Supplementary Tables 1–3). A study by Bawin et al. (2014) revealed that the attraction of *T. absoluta* is mediated by the volatile signature of the host plant. Tomato leaf odors usually include volatile terpenoid compounds (Proffit et al., 2011; Anastasaki et al., 2018), which may have an influence on the preference of *T. absoluta* to tomatoes than eggplants. These observations demonstrated the essential role of plant volatiles in *T. absoluta* host-finding behavior. For instance, nerolidol and other compounds have been reported by earlier researchers to be present in tomatoes or induced upon infestation by *T. absoluta* (Supplementary Tables 1–3) (Silva et al., 2018). Most of these compounds have been demonstrated to repel the tomato borer, *T. absoluta* (Campolo et al., 2017).

Intriguingly, several VOCs including four terpenes (nerolidol 2; beta-Cyclocitral; 1,3-cyclohexadiene-1-carboxaldehyde, 2,6,6-trimethyl, and beta.-iso-methyl ionone), one ketone (cyclohexanone, 2,2,6-trimethyl), one heterocyclic compound (furan, 2-pentyl), two ester [2-butenoic acid, 3-hexenyl ester, (E,Z) and (E)-hex-3-enyl (E)-2-methylbut-2-enoate], two aldehydes (benzaldehyde and 2-Ethylbenzaldehyde), and two alcohols (cyclohexanol, 2,6-dimethyl and 1-octen-3-ol) were naturally emitted by tomatoes, which may serve as attractants and stimulants to *T. absoluta* (Supplementary Table 1). These compounds may have contributed to the *T. absoluta* preference for tomato and thus, could be exploited in the pull-and-push strategy of pest management in solanaceous species (Cook et al., 2007).

Conversely, three DAVOCs were emitted by eggplants upon infestation by *T. absoluta*, which were nerolidol 2 (terpene), 1,3-cyclopentadiene, 5,5-dimethyl-1,2-dipropyl (olefin), and 2-butenoic acid, 3-hexenyl ester (E,Z) (ester) (Supplementary Table 2). These compounds have been demonstrated to be induced upon wounding or infestation in some plants and may contribute to repel *T. absoluta* survival, multiplication, and damage in eggplants. For instance, 1,4,7, cyclododecatriene, 1,5,9,9-tetramethyl was identified to have antimicrobial activity.

Among the primary/secondary metabolites differentially detected in this study, terpene compounds (Supplementary Tables 4–6; Supplementary Figures 2A,D) were in higher abundance in tomatoes than eggplants (either control or *T. absoluta*-infested). The abundance of terpene compounds in tomatoes are in consonance, which reported that most of these compounds are used by insect pests to recognize the host plants as attractants and feeding stimulants. Three typical examples, namely, capsianoside IV, capsianoside II, and 6'-O-D-glucosylsweroside, may serve as attractants aiding *T. absoluta* larvae to identify a susceptible host (Robert et al., 2012, 2013).

Strikingly, two flavonoid compounds [6-hydroxykaempferol-3-O-rutin-6-O-glucoside and quercetin-3-O-apiosyl (1 → 2) galactoside] and other classes of compounds were induced in eggplants upon *T. absoluta* infestation. These flavonoid compounds have been reported to have negative effects on insects and pathogens in several crops (Simmonds, 2001). Plants emit several chemical compounds both to deter and attract insects; in some cases, the natural predators of herbivores. Among them, flavonoids have been documented to modulate oviposition and feeding. In this study, naringenin, quercetin-3-O-rutinoside, and other flavonoids have been found to be accumulated higher in eggplants or were completely absent in tomatoes. These compounds have been shown to prevent insects from laying eggs and deter *Pieris rapae*. Apigenin-6-C-rhamnoside, a flavonoid compound, was detected only in Tomato-Control. This compound has been reported to alter the palatability of plants to pathogens (Simmonds and Stevenson, 2001). On the contrary, this compound may work in the opposite direction compared with earlier reports (Simmonds and Stevenson, 2001). Therefore, the occurrence and abundance of flavonoid compounds under control and *T. absoluta-*infested Eggplant_vs_Tomato may have influenced the *T. absoluta* preference for tomato as a host.

The results of the EAG experiments indicated that the antennae of *T. absoluta* respond to all VOCs (Figure 7). This experiment suggested that a minute quantity of VOCs can act as an attractant or repellent to *T. absoluta* to either tomatoes or eggplants. These compounds have been reported in previous studies to repel or attract *T. absoluta* in tomatoes/eggplants (Bawin et al., 2014; Anastasaki et al., 2018). Thus, these compounds detected by the EAG experiment may contribute to the altering of the *T. absoluta* preference for host plants.

## Conclusions

The suitability of tomatoes as hosts for *T. absoluta* invasion is much higher than that for eggplants based on the results of this study, which compared the metabolomes and volatilomes from both plants. The key metabolites and volatiles that are vital in modulating *T. absoluta* damage in tomatoes have also been reported, and future research could focus on them. These compounds could be validated for use as selection signatures in screening tomato accessions against *T. absoluta* in our future studies. The results provided in this study provide a foundation that will allow us to develop effective biological control methods of *T. absoluta* in cultivated tomatoes and other *Solanum* crops.

## Methods

### Plant Materials and Growth Conditions

The tomato variety Zhefen 202 and eggplant variety Zheqie No.1 were used in this study. The plant materials were obtained from the Zhejiang Academy of Agricultural Science, and no permissions are necessary to work on such materials. Using tomato as the host plant, a population of the South American tomato pinworm, *T. absoluta* (Meyrick), was collected in Ili, Xinjiang (China) in 2018 and maintained continuously in an artificial climate chamber [25°C, relative humidity (60%), 16 h light (L): 8 h dark (D)]. The plants were grown in an insect-free greenhouse at the Zhejiang Academy of Agricultural Science research station (day/night temperature 24/20°C, relative humidity 60%, 16 L: 8 D) under controlled conditions. Following the cleaning and germination of tomato and eggplant seeds, the seedlings were cultivated in coconut bran to produce fruits. When the seedlings reached the two-leaf stage, they were transplanted into a plastic flowerpot with a diameter of 10 cm and a height of 9 cm, where they were allowed to continue to grow. Each pot included a single plant, which was watered once every 2 days during the growing season. For each pot of host plants, 2 g of an OMEX 18-18-18 (OMEX, UK) macro-elements water-soluble fertilizer (containing 18 macro-elements) were administered to encourage the growth of the host plants. The host plants were not exposed to any pesticides during the experiment. Furthermore, *T. absoluta* larvae were inoculated into eggplants at the five-leaf stage and tomato plants with 20–50 normal leaves, which were both grown in a greenhouse.

### Preparation of Samples for Metabolite Extraction

Using a mixer mill MM400 with zirconia beads (Retsch GmbH, Germany) with a 15-mm size and running at 30 Hz for 1.5 min, 3 g of each of the samples from the four treatments (Tomato-Control, Tomato-*T. absoluta* infested, Eggplant-Control, and Eggplant-*T. absoluta* infested) with three biological replicates (thus, 12 samples) were crushed into powder after vacuum freeze-drying Retsch GmbH, Germany. For the extraction of the metabolites, a 100-mg sample of the powder was further extracted overnight at 4°C in 1 ml of 70% aqueous methanol. After 10 min of centrifugation at 10,000 × g, the extract was removed. The extraction procedure was done, and the extracts were transferred to a fresh tube for LC-MS analysis. In order to validate the data, quality control (QC) analyses (mixing extracts for insertion into every three samples) were done. MetWare Biotechnology Company completed the analysis in accordance with previous protocols (Wang et al., 2019).

### Identification and Quantitation of Metabolites

Metabolite profiling was undertaken with the use of a self-built database (http://www.metware.cn) developed by MetWare Biotechnology Co. Ltd. (Wuhan, China) (Li et al., 2013; Melvin et al., 2015). With the help of secondary spectrum information, the metabolites were qualitatively evaluated. Besides, metabolite quantification was carried out utilizing the triple quadruple-bar mass spectrometry in the multi-reaction monitoring (MRM) mode. A principal components analysis (PCA) plot was used to visualize the differences and similarities between and among samples. Using partial least squares-discriminant analysis (PLS-DA) with a log2FC 1 threshold and VIP 1, we were able to identify the DAMs. An analysis of the function of DAMs was carried out in the Kyoto Encyclopedia of Genes and Genomes (KEGG) database (Kanehisa et al., 2016).

### Determination of Host Plant Volatiles by GC-MS

Five healthy and insect-induced host plants (tomato and eggplant) were selected. After removing the veins, samples were lyophilized in liquid nitrogen (LN) and stored in a −80°C refrigerator. Samples were later pulverized in LN and vortexed to mix evenly. The samples were put in a headspace bottle with fully automatic HS-SPME (Lee et al., 2007). The GC-MS was used to identify terpenoids, benzene ring types and phenylpropanoids, fatty acid derivatives, and other volatiles. The volatile content was determined by the headspace collection method or extraction method. The SPME readings were taken at a 250°C aging temperature; 5-min aging time; 60°C heating temperature; 10-min heating time; 20-min adsorption time; 5-min desorption time; 5-min aging time after sample injection. The original data file obtained by GC-MS analysis was first extracted using the MassHunter software (Agilent, CA, USA).

### Metabolites Determination

Extracts from the samples were analyzed using an LC-ESI-MS/MS system (HPLC, Shim-pack UFLC SHIMADZU CBM30A system, Kyoto, Japan; MS, Applied Biosystems 6,500 Q TRAP, San Diego, CA, USA). It was possible to link the HPLC effluent to an electrospray ionization (ESI)-triple quadrupole-linear ion trap–mass spectrometry/mass spectrometry apparatus instead (Ap-plied Biosystems 4,500 Q TRAP, San Diego, CA, USA). The analytical conditions were modified from Wang et al. (2010) and Hsu et al. (2017). Using multiple-reaction monitoring (MRM) (Hsu et al., 2017) and the MetWare MWDB database, the researchers were able to quantify metabolites using their usual metabolic operating methods (Chen et al., 2009, 2013).

### Electrophysiological Responses of *Tuta absoluta* to DAVOCs

There were 35 DAVOCs identified between treatments, which were selected for electroantennographic tests, including 16 increased VOCs (benzyl alcohol, 2-methoxyestradiol, phenylethyl alcohol, eugenol, 2-isopropyl-3-methoxypyrazine, 2-isobutyl-3-methoxypyrazine, isoamyl acetate, 2,4-dichlorophenol, β-pinene, geranyl acetone, isopropyl isocyanate, 4-propylphenol, tyramine, 2,4-quinolinediol, phytone, and o-nitrophenol) and five decreased VOCs (trans,cis-2,6-non-adienal, trans,trans-2,4-non-adienal, hexadecane, γ-caprolactone, and 2,2,4,4,6,8,8-heptamethylnonane) of tomato compared with eggplant; five increased VOCs (benzaldehyde, 2-pentylfuran, beta-cyclocitral, 1-octen-3-ol, and 2-Ethylbenzaldehyde) and three decreased VOCs [(+)-alpha-pinene, 1-heptanol and 1-nonanol] of tomato after infestation; six increased VOCs (furfuryl methyl disulfide, phenylacetaldehyde, hexyl acetate, 4-ethoxybenzaldehyde, maleimide, and (Z)-2-penten-1-ol) and two decreased VOCs (1-heptanol and 1-nonanol) of eggplant after infestation.

The volatiles were tested for their antennal responses by *T. absoluta*, which was detected utilizing the EAG detection system (Stimulus Air Controller CS-55 and SYNTECH IDIC-2; Syntech, Hilversum, the Netherlands). Using three different concentrations (1, 10, 100, and 1,000 g/ml) of the standard chemicals, 10 ml of each were applied to a filter paper strip for analysis. After allowing the solvent to evaporate for 2 min, the strip was placed in a Pasteur pipette, and the process was repeated. Similarly, to the case of indole, the control stimulus was paraffin oil (10 m). The application of 2-s puffs of air through a Pasteur pipette containing the filter paper holding the stimuli resulted in test stimulations being performed. During the test, puffs of the test stimuli were administered at 1-min intervals randomly. Antennal preparations were monitored using puffs of paraffin oil administered at the start and end of each experiment and between groups of five to six compounds to ensure that they were in good condition. The antennal responses of at least three females to varying doses of substances were recorded in the laboratory. When compared with the control, the EAG reactions were normalized and reported as a percentage of the response to paraffin oil.

### Data Analyses

A QC analysis was undertaken to ensure that the data was reliable before proceeding with the overall analyses. The QC sample was created by combining sample extracts for insertion into every three samples to monitor changes in the results of several analyses. The analyses of the samples were performed using the Analyst software (version 1.6.1; AB Sciex, Canada), which loaded data matrices with the intensity of the metabolite characteristics from the samples. The PLS-DA was used to maximize the differences in the metabolomes between the two sample pairs in this study. The relative relevance of each metabolite to the PLS-DA model was determined by utilizing the VIP as a parameter to test the hypothesis. Metabolites with a VIP ≥1 and fold change ≥2 or fold change ≤0.5 were considered as differential metabolites for group discrimination (Chong et al., 2018). The PCA and Ward's hierarchical clustering heatmap were performed using R software (version 3.3.2; www.r-project.org) (Chong and Xia, 2019). Consequently, a metabolic pathway was constructed according to KEGG (http://www.genome.jp/kegg/) (Goto, 2000). Furthermore, a pathway analysis was performed using MetaboAnalyst (http://www.metaboanalyst.ca/) based on the change in metabolite concentration compared with the corresponding controls (Chong et al., 2018). Data obtained from EAG were subjected to an ANOVA, and means separation was done by Turkey's Highest Significant Difference (version 8, GraphPad Software, San Diego, CA, USA, www.graphpad.com) at *P* ≤ 0.05.

## Data Availability Statement

The original contributions presented in the study are included in the article/Supplementary Material, further inquiries can be directed to the corresponding authors.

## Author Contributions

LC, XL, YH, and YL: conceptualization. LC and XL: methodology and writing–original draft preparation. LC, XL, JZ, and TH: software. ZZ and MH: validation. LC, XL, YW, SZ, and XR: formal analysis. LC, XL, JZ, TH, JH, ZZ, MH, SZ, and XR: investigation. YH and YL: writing—review and editing, project administration, and funding acquisition. All authors have read and approved the final version of the manuscript.

## Funding

This research was funded by the Primary Research & Development Plan of Lishui (No. 2020ZDYF02), the National Key R&D Program of China (No. 22017YFC1200605), Project of State Key Laboratory for Managing Biotic and Chemical Threats to the Quality and Safety of Agro-products (No. 2010DS700124-ZZ2015), and the Fujian Science and Technology Special Project (No. 22017NZ0003-1-6). The funders had no role in the study design, data collection and analysis, decision to publish, or preparation of the manuscript.

## Conflict of Interest

The authors declare that the research was conducted in the absence of any commercial or financial relationships that could be construed as a potential conflict of interest.

## Publisher's Note

All claims expressed in this article are solely those of the authors and do not necessarily represent those of their affiliated organizations, or those of the publisher, the editors and the reviewers. Any product that may be evaluated in this article, or claim that may be made by its manufacturer, is not guaranteed or endorsed by the publisher.

## Abbreviations

ANOVA: analysis of variance
DAVOC: differentially accumulated volatile organic compounds
EAG: electroantennogram
GC-MS: gas chromatography-mass spectrometry
PCA: Principal component analysis
PLS-DA: partial least squares discriminant analysis
QC: quality control
UPLC-MS/MS: ultra-performance liquid chromatography-tandem mass spectrometry
VIP: variable importance in projection
VOC: volatile organic compounds.
