# Supplementary material for: Comprehensive Metabolome and Volatilome Analyses in Eggplant and Tomato Reveal Their Differential Responses to Tuta absoluta Infestation
Source: Front Plant Sci. 2021 Nov 3;12:757230. doi: 10.3389/fpls.2021.757230 (PMC8597266; doi:10.3389/fpls.2021.757230)
Supplement: Supplementary file 1 [file Data_Sheet_1.zip › Supplementary Figure S1.DOCX]

**
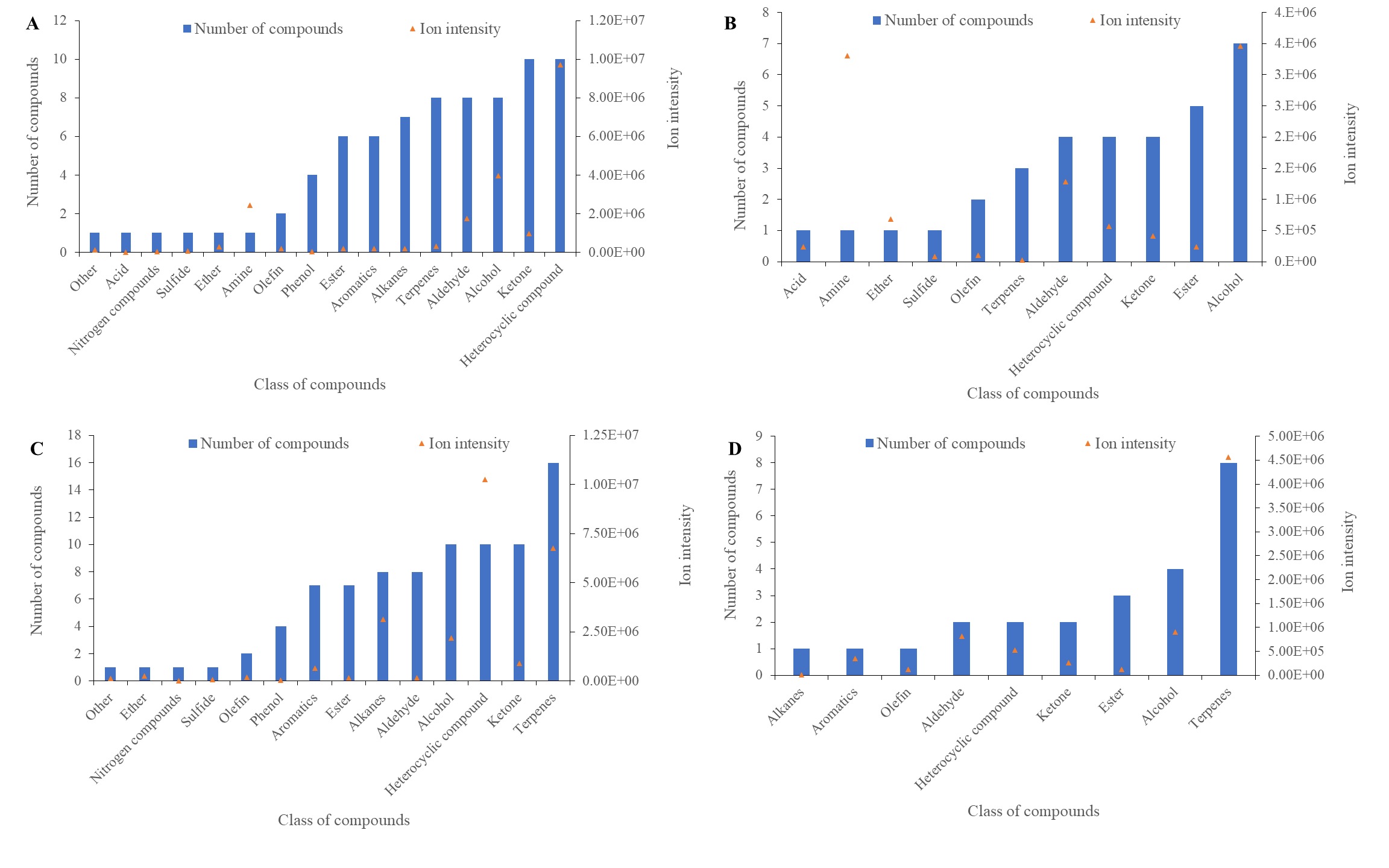
**

**Supplementary Figure S1.** Classes of volatile organic compounds (differentially accumulated) detected among the four individual groups ( Y-axis on right hand side (primary axis) represents number of unique compounds detected in each class (blue bar) and Y-axis on left hand side (secondary axis) represents summation of ion intensities of unique compounds detected in each class (chocolate triangle). **(A).** Eggplant-Control.  **(B).** Eggplant-*Tuta absoluta* infested. **(C).** Tomato-Control.  **(D).** Tomato-*Tuta absoluta* infested.
